# Association of monocyte-lymphocyte ratio and proliferative diabetic retinopathy in the U.S. population with type 2 diabetes

**DOI:** 10.1186/s12967-022-03425-4

**Published:** 2022-05-13

**Authors:** Huan Wang, Zhen Guo, Yu Xu

**Affiliations:** Tianjin Children’s Hospital, Tianjin, 300134 China

**Keywords:** Proliferative diabetic retinopathy, Monocyte-lymphocyte ratio, Type 2 Diabetes, NHANES

## Abstract

**Objective:**

Diabetic retinopathy (DR), especially proliferative diabetic retinopathy (PDR) is a common cause of blindness and visual impairment. Early prediction of its occurrence and progression is important to improved patient outcomes. Inflammation-related markers may play important roles, and the monocyte-lymphocyte ratio (MLR) can act as a novel inflammatory marker. However, the association between MLR and PDR remains unclear. The aim of the present study was to investigate the association between MLR and PDR in the U.S. population with type 2 diabetes (T2D) based on DR data from NHANES in 2005–2008.

**Methods:**

This cross-sectional study was conducted in the National Health and Nutrition Examination Survey (NHANES) from 2005 to 2008. DR was defined by the criteria of the Early Treatment for Diabetic Retinopathy Study based on nonmydriatic fundus photography. The MLR is the monocyte count/lymphocyte count. The lymphocyte count and monocyte count can be obtained directly from laboratory data files. Logistic regression was used to explore the association between MLR and PDR. Stratified analyses were also conducted according to age, sex, hemoglobin, and glycated hemoglobin categories. We applied the duration of diabetes with multiple imputations of missing data.

**Results:**

A total of 367 participants were included, among whom the PDR prevalence was 7% (27/367). Multivariate regression models revealed that PDR was significantly associated with 0.1 unit increase in MLR (adjusted OR = 1.46, 95% CI: 1.08−1.96) after all covariates were adjusted. In the subgroup analysis, effect size of MLR on the presence of PDR in subgroups were stable (all P values > 0.05).

**Conclusions:**

MLR was significantly associated with PDR in participants with T2D. Assessing the MLR might be a valuable part of follow-up visits for patients with T2D.

**Graphical Abstract:**

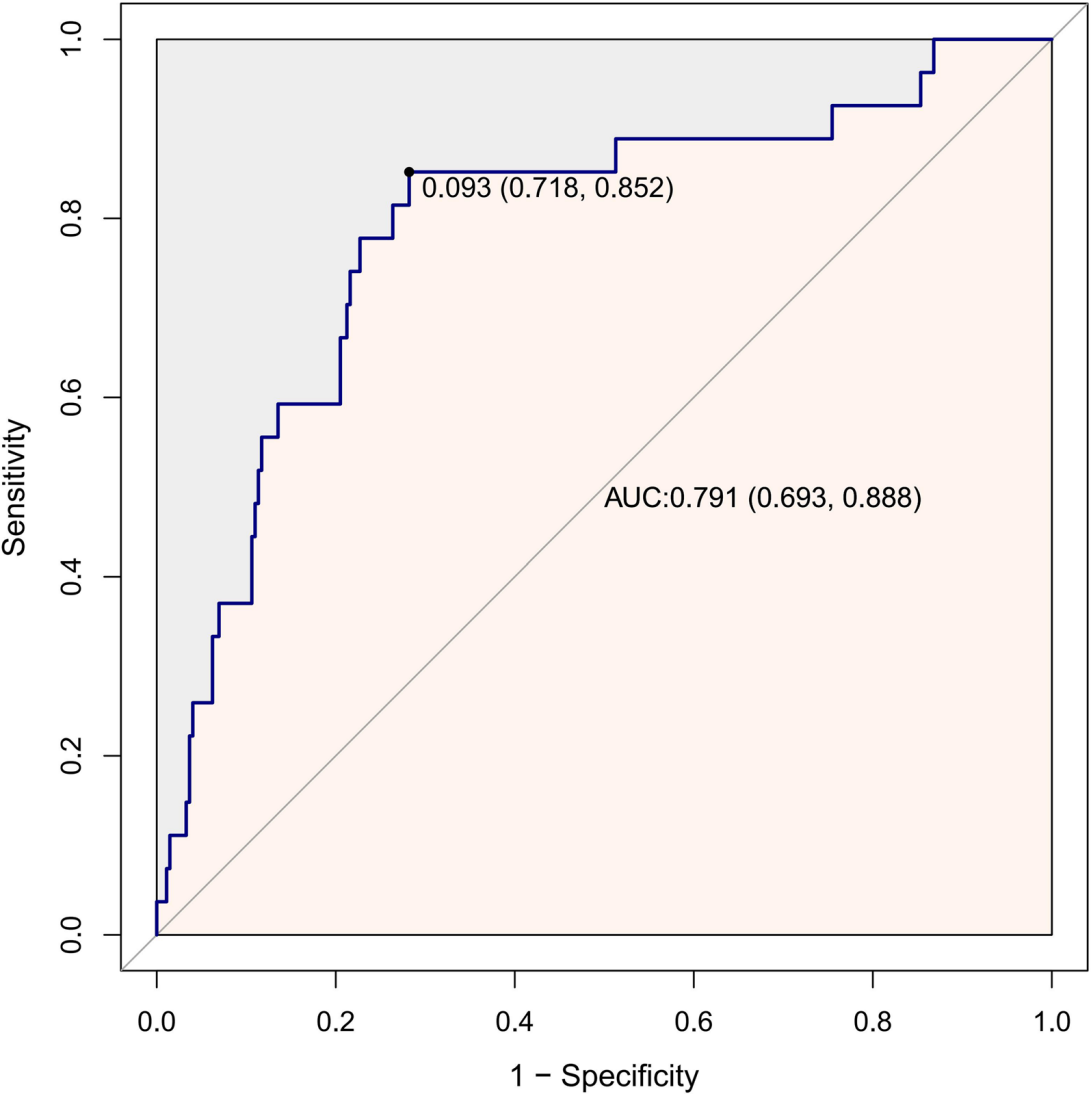

**Supplementary Information:**

The online version contains supplementary material available at 10.1186/s12967-022-03425-4.

## Introduction

Diabetic retinopathy (DR) is a leading cause of blindness, contributing to 2.6% and 1.9% of visual impairment and blindness worldwide [1], respectively in western countries, 33% of patients with diabetes have DR [2]. Early identification of microvascular complication risks provides an opportunity to delay or stop disease onset [3].

Type 2 diabetes is chronic inflammation by increasing insulin resistance and disturbed glucose metabolism. DR as a vascular and neurodegenerative disease occurs after some years of poorly controlled diabetes [4]. Leukocyte activation can cause repeated episodes of capillary occlusion and, progressive retinal ischemia. DR is induced by low grade and persistent leukocyte activation [5]. The upregulation of vascular endothelial growth factor (VEGF) and its receptors is intimately associated with retinopathy progression. VEGF plays a major role in the development of DR [6, 7]. Several biomarkers can reflect the presence of microvascular complications [8] and are also associated with an increased risk of retinopathy [9].

The amount of evidence indicating that several inflammatory markers are associated with an increased risk of DR [10] and that inflammation-related markers play important roles in the prediction and disease assessment of DR has been increasing. The monocyte-lymphocyte ratio (MLR) is a novel inflammatory marker that plays an important role in the prediction and prognosis of some inflammation-related diseases, such as cancer, cardiovascular diseases and DR [11]. Previous studies have shown that white blood cell (WBC) subtypes are closely associated with the inflammatory state of DR [12]. We speculated that MLR may play an important role in the development and progression of DR and be highly significant in PDR patients. However, the association between MLR and PDR remains unclear. Thus, the aim of this study was to explore the clinical and predictive significance of MLR in T2D patients with PDR.

## Materials and methods

### Study design and participants

The National Health and Nutrition Examination Survey (NHANES) is conducted by the National Center for Health Statistics (NCHS), in which non-institutionalized civilians in the U.S. can participate. All participants underwent comprehensive measurements and standardized interview questionnaires, such as physical and laboratory examinations and socioeconomic, demographic, and health-related questions, respectively.

In our study, we used public data from two NHANES cycles (2005–2006 and, 2007–2008). More information regarding the data is available on the NHANES website (www.cdc.gov/nchs/nhanes/). Survey participants were invited to undergo including visual acuity testing and blood testing. Retinal photographs were obtained for participants 40 years or older. Self-reported basic sociodemographic data and medical history were provided by home interviews.

This study was approved by the Institutional Review Board of the NCHS and conducted in accordance with the tenets of the Declaration of Helsinki. All participants provided informed consent before being examined.

## Study variables and outcome

MLR is the monocyte count/lymphocyte count. Both these values can be obtained directly from laboratory data files. The neutrophil count was calculated from the WBC and neutrophil percentages.

The T2D was defined by the American Diabetes Association criteria [13] and a self-report questionnaire. Participants who fulfilled the following criteria were identified as T2D [14]: (1) Glycated hemoglobin (HbA1c) ≥ 6.5%, (2) Fasting plasma glucose (FPG) ≥ 7 mmol/L; (3) during an oral glucose tolerance test, 2-hplasma glucose ≥ 11.1 mmol/L; (4) self-report questionnaire data indicating physician diagnosis of diabetes; and (5) lower blood glucose by current use of insulin or diabetes pill.

DR [15] was defined by the presence of hemorrhages, hard exudates, cotton wool spots, microaneurysms, venous beading, intraretinal microvascular abnormalities, and new retinal vessels based on the severity scale of the Early Treatment for Diabetic Retinopathy Study. Non-mydriatic fundus photography (TRC-NW6S; Topcon, Tokyo, Japan) was used to measure the level of retinopathy in the worse eye. The grades were categorized into no DR, non-proliferative DR, and proliferative DR. Detailed information is listed in the Digital Grading Protocol of the NHANES.

Other covariates included sex (male or female), age, race (non-Hispanic white, non-Hispanic Black, Mexican American, other Hispanic, and other), marital status (married, unmarried, and other), body mass index (BMI) (< 25.0, 25.0–29.9 and ≥ 30.0 kg/m^2^), HbA1C (< 6.5%, ≥ 6.5%), HGB, C-reactive protein (CRP), high-density lipoprotein cholesterol(HDL) and Total cholesterol. Smoking status [14] was categorized as current smokers, former smokers, and never smokers. Participants who had smoked more than 100 cigarettes in the past and reported smoking either some days or every day at the time of the interview were considered to be current smokers, who had smoked more than 100 cigarettes during their lifetime but did not smoke currently were considered former smokers, and who reported not having smoked even 100 cigarettes during their lifetime were considered never smokers. The duration of diabetes was calculated using the reported age at screening minus the age of the subject when they were first informed that they had diabetes. Family history of diabetes was determined using the participant’s answer to the following question: ‘Including those living and deceased, were any of your biological relatives, that is, blood relatives, including grandparents, parents, brothers, and sisters, ever told by a health professional that they had diabetes?’. CRP [16] was quantified by latex-enhanced nephelometry. Respondents with CRP below the lowest sensitivity were assigned to 0.01 or 0.02 mg/dL.

### Statistical analysis

All analyses were performed using the statistical software packages R (http://www.R-project.org, The R Foundation) and Free Statistics software version 1.3. The differences in continuous and categorical variables were investigated using the independent and chi-squared tests, respectively. These logistic regression models were used to determine the relationship between MLR and the presence of PDR. Model 1 was unadjusted, model 2 was adjusted for age, sex, race, HGB, and duration of diabetes. Subgroup analysis was used to examine the relationship between MLR and PDR according to the age, sex, HGB category (bisection), and HbA1C category (< 6.5, ≥ 6.5). The test for interaction in the logistic regression model was used to compare odds ratios (ORs) between the analyzed subgroups. For all analyses, missing values of the duration of diabetes were 67 (367). The percentages of missing values were lower than 20%. We imputed missing data of the covariates by using multiple imputations. Five datasets were created and analysed together.

## Results

### Study population characteristics

**Fig. 1:**
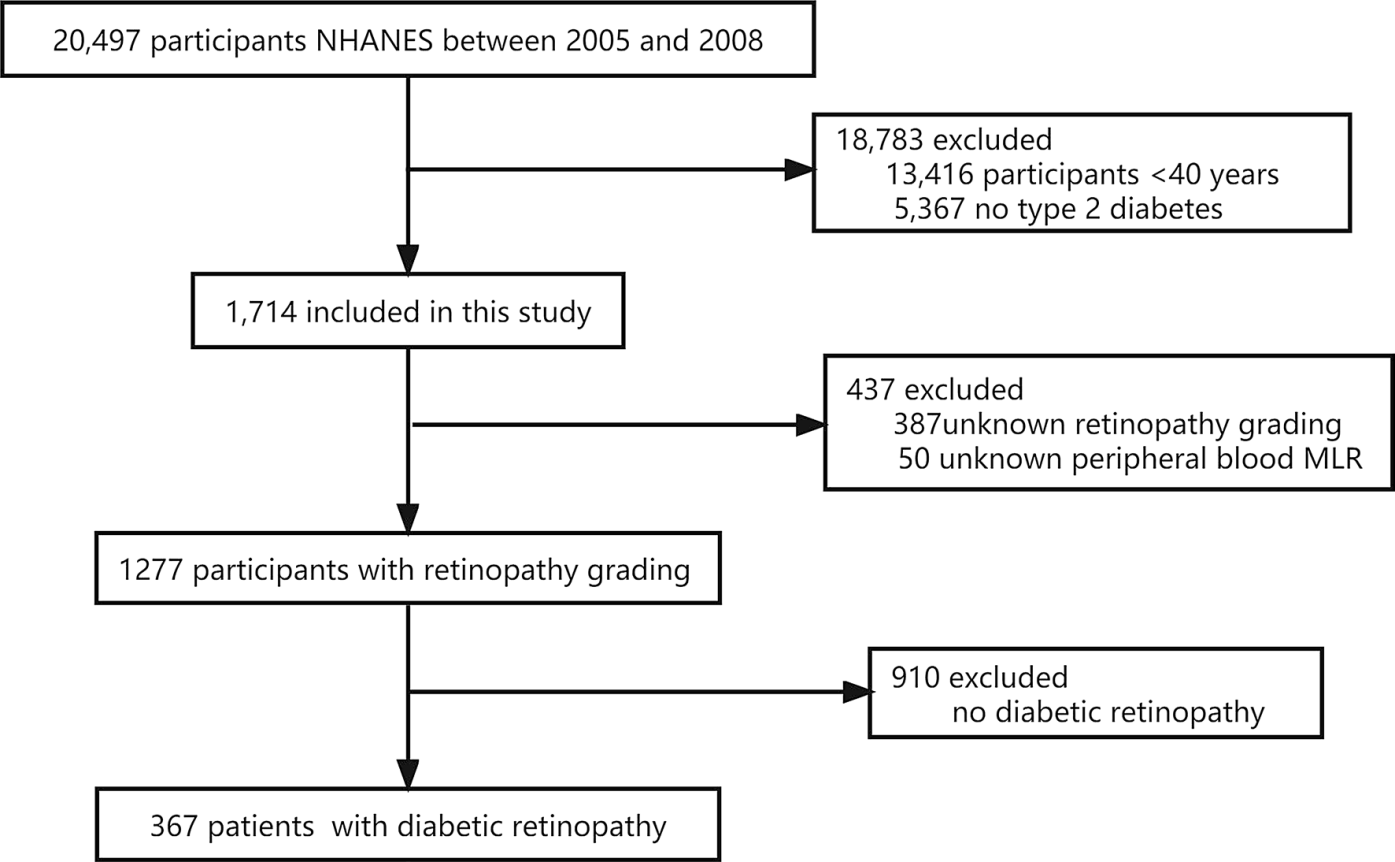
Schematic overview for patient identification. NHANES, National Health and Nutrition Examination Survey

Two cycles of NHANES, 2005–2006 and 2007–2008, were used in our study. We identified 20,497 potential participants. 7081 adults (≥ 40 years old) completed the interview and the MEC examination. 5367 participants with no diabetes were excluded. Participants with missing data on retinopathy grading (n = 387) and peripheral blood MLR (n = 50) were excluded. After excluding participants with no DR (n = 910), a total of 367 participants were included in the analysis. The flow chart of the inclusion and exclusion criteria is depicted in Fig. 1. Table 1 shows the demographic, socioeconomic, comorbidity, and baseline characteristics of PDR and non-proliferative diabetic retinopathy (NPDR). PDR was detected in 27 participants (7%). Significant differences in HGB and duration of diabetes were observed between the PDR and NPDR groups (all P-values < 0.05). Lower HGB levels was observed in the PDR group (P < 0.001). Subjects with PDR had the longest duration of diabetes (21.6 years, P = 0.002) compared to those with NPDR (14.8 years).

**Table 1:** Baseline characteristics of participants

Characteristics	Total	NPDR	PDR	
n	367	340	27	p
Age (years)	63.8 ± 10.8	63.9 ± 11.0	63.0 ± 7.8	0.691
*Sex, n (%)*				0.242
Male	196 (53.4)	185 (54.4)	11 (40.7)	
Female	171 (46.6)	155 (45.6)	16 (59.3)	
*Race/ethnicity, n (%)*				0.104
Non-Hispanic white	133 (36.2)	128 (37.6)	5 (18.5)	
Non-Hispanic black	121 (33.0)	109 (32.1)	12 (44.4)	
Mexican American	76 (20.7)	71 (20.9)	5 (18.5)	
Other	37 (10.1)	32 (9.4)	5 (18.5)	
*Marriage, n (%)*				0.245
Married	225 (61.3)	212 (62.4)	13 (48.1)	
Unmarried	21 ( 5.7)	19 (5.6)	2 (7.4)	
Other	121 (33.0)	109 (32.1)	12 (44.4)	
*BMI, n (%)*				0.161
Underweight/normal	46 (12.6)	41 (12.1)	5 (19.2)	
Overweight	129 (35.2)	124 (36.5)	5 (19.2)	
Obese	191 (52.2)	175 (51.5)	16 (61.5)	
*Smoking status, n (%)*				0.171
Current smokers	58 (15.8)	57 (16.8)	1 (3.7)	
Former smokers	122 (33.2)	112 (32.9)	10 (37.0)	
Never smokers	187 (51.0)	171 (50.3)	16 (59.3)	
*HbA1C, n (%)*				0.351
< 6.5	91 (24.9)	87 (25.7)	4 (15.4)	
> = 6.5	274 (75.1)	252 (74.3)	22 (84.6)	
HGB(g/dL)	13.7 ± 1.7	13.8 ± 1.7	12.7 ± 1.4	< 0.001
CRP	0.6 ± 1.1	0.6 ± 1.1	0.8 ± 0.9	0.388
VitD (nmol/L)	53.0 ± 22.1	52.9 ± 21.7	54.0 ± 27.4	0.803
HDL (mmol/L)	1.3 ± 0.4	1.3 ± 0.4	1.3 ± 0.4	0.617
Total cholesterol(mmol/L)	4.9 ± 1.3	4.9 ± 1.3	5.2 ± 1.2	0.175
Family history of diabetes, n (%)	237 (64.6)	216 (63.5)	21 (77.8)	0.354
Duration of diabetes (years)	15.4 ± 11.0	14.8 ± 11.1	21.6 ± 8.8	0.002

NPDR, non-proliferative diabetic retinopathy; PDR, proliferative diabetic retinopathy; BMI, body mass index; HGB, hemoglobin; CRP, C-reactive protein; Vitamin D, Vit D; HDL, high-density lipoprotein cholesterol

### Factors associated with PDR

Regression analysis was performed to identify factors in the entire study population that were associated with PDR. The results of univariate ordinal regression analysis indicated that PIR, race, HGB, and duration of diabetes were positively associated with PDR (all P < 0.05, Table 2).

**Table 2:** Univariate analysis for the presence of PDR

Characteristics		
n	OR (95% CI)	P
Age (years)	0.99 (0.96–1.03)	0.690
*Sex, n (%)*		0.175
Male	1	
Female	1.74 (0.78–3.85)	
*Race/ethnicity, n (%)*		
Non-Hispanic white	1	
Non-Hispanic black	2.82 (0.96–8.25)	0.059
Mexican American	1.80 (0.5–6.44)	0.364
Other	4.00 (1.09–14.66)	0.036
*Marriage, n (%)*		
Married	1	
Unmarried	1.72 (0.36–8.18)	0.497
Other	1.8 (0.79 ~ 4.07)	0.161
*BMI*		
Underweight/normal	1	
Overweight	0.33 (0.09–1.2)	0.092
Obese	0.75 (0.26 ~ 2.16)	0.594
*Smoking status, n (%)*		
Current smoker	1	
Former smoker	5.09 (0.64–40.73)	0.125
Never smoker	5.33 (0.69–41.1)	0.108
VitD (nmol/L)	1.00 (0.98–1.02)	0.803
HGB(g/dL)	0.68 (0.54–0.86)	0.001
CRP	1.10 (0.87–1.39)	0.414
HDL (mmol/L)	1.31 (0.45–3.78)	0.616
Total cholesterol(mmol/L)	1.22 (0.91–1.63)	0.176
Family history of diabetes, n (%)	0.53 (0.21–1.36)	0.186
Duration of diabetes (years)	1.04 (1.01–1.07)	0.004

OR, odds ratio; CI, confidence interval; BMI, body mass index; Vitamin D, Vit D; HGB, hemoglobin; CRP, C-reactive protein; HDL, high-density lipoprotein cholesterol

### Association between MLR and the presence of PDR

Table 3 shows the odds ratios and 95% confidence intervals (CIs) for the presence of PDR determined by MLR. In the non-adjusted model, MLR was significantly associated with the presence of PDR (OR = 1.40, 95% CI: 1.11–1.75). Each 0.1 unit increase in MLR was associated with 40% increase in the presence of PDR. In the multivariate regression models, after adjusting for age, sex, and race/ethnicity, the odds ratio was 1.59 (1.22–2.07); after adjusting for age, sex, race/ethnicity, HGB, and duration of diabetes, the odds ratio was 1.46 (1.08–1.96).

**Table 3:** Association between MLR and the presence of PDR

	PDR (n = 27)					
	Mode l1		Model 2		Mode l3	
	OR (95% CI)	P	OR (95% CI)	P	OR (95% CI)	P
MLR*10	1.4 (1.11–1.75)	0.004	1.59 (1.22–2.07)	0.001	1.46 (1.08–1.96)	0.014

Adjusted covariates: Model 1: unadjusted; Model 2: adjusted by age, sex, race; Model 3: Model 2 + HGB, duration of diabetes. MLR, monocyte-lymphocyte ratio; PDR, proliferative diabetic retinopathy; OR, odds ratio; CI, confidence interval; HGB, hemoglobin

In addition, the missing values of the duration of diabetes were 67 (367). We imputed missing data of the covariates by using multiple imputations. Five datasets were created and analysed together (Additional file 1: Tables S1–S5).

### Subgroup analyses of factors influencing the association between MLR and the presence of PDR

**Fig. 2:**
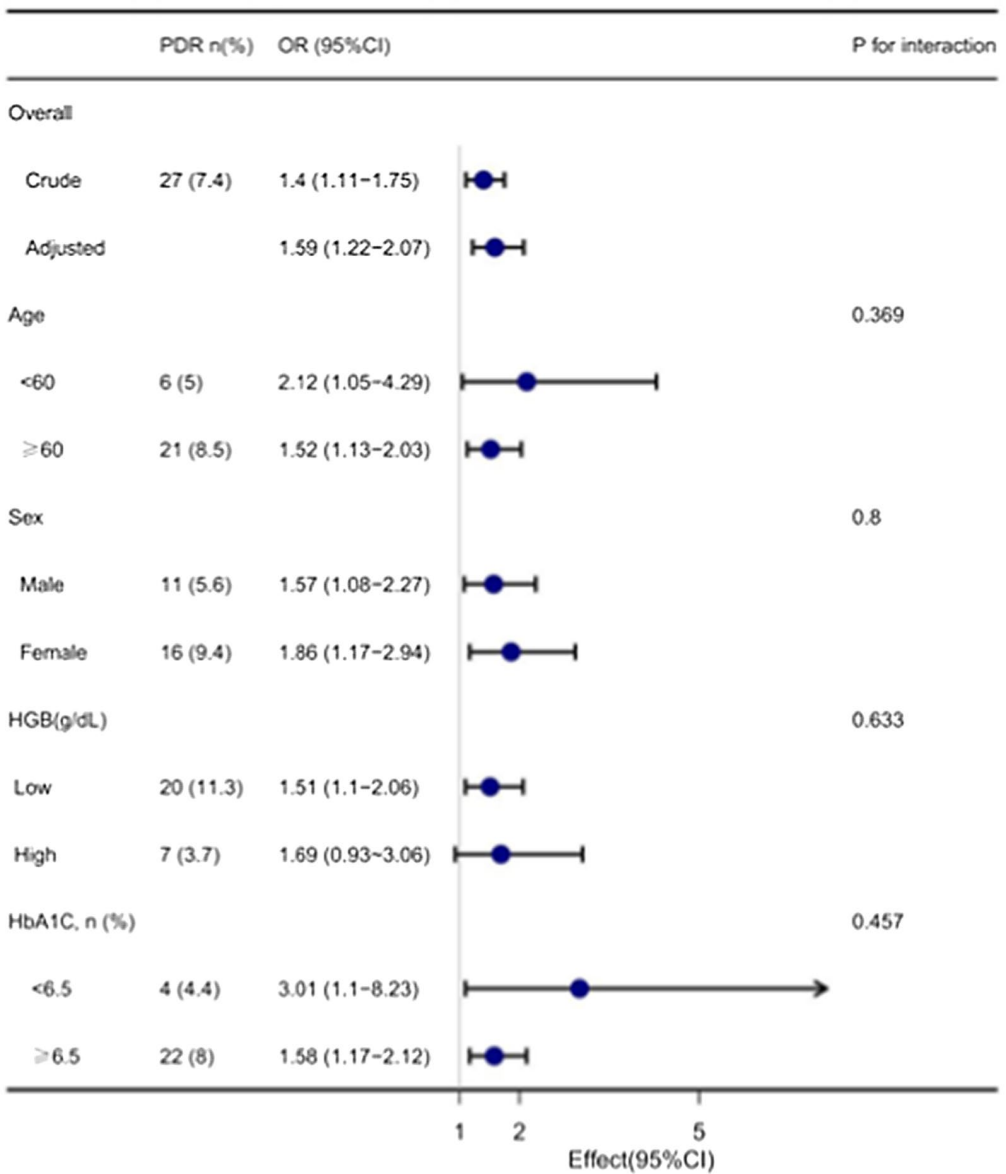
Effect size of MLR on the presence of PDR in the age, sex, HGB, HbA1c subgroup. OR, odds ratio; CI, confidence interval; MLR, monocyte-lymphocyte ratio; PDR, proliferative diabetic retinopathy; HGB, hemoglobin

In the subgroup analysis stratified by age, sex, HGB category (bisection), and HbA1C category (< 6.5, ≥ 6.5), the association between MLR and the presence of PDR was explored in Fig. 2. Effect size of MLR on the presence of PDR in subgroups were stable. The interaction analysis of MLR and age (p for interaction = 0.369), MLR and sex (p for interaction = 0.800), MLR and HGB (p for interaction = 0.633), and MLR and HbA1C (p for interaction = 0.457) in regard to the presence of PDR were not significant.

## Discussion

We used the NHANES database to conduct the present study. To our knowledge, our results were the first to show that MLR increased as the incidence of PDR increased. There was a significant correlation between MLR and PDR. The MLR was associated with the prevalence of PDR after other confounding factors were adjusted for. This indicates that MLR might be used to predict the occurrence and progression of PDR.

Increasing amounts of evidence have emerged showing that chronic inflammation plays a dominant role in the development of DR [17]. Grossman et al. pointed out that the WBC, granulocytes, and monocyte levels, not those of lymphocytes, were higher in normoglycemic subjects than in subjects with diabetes [18]. Ji et al. reported that the MLR or lymphocyte to monocyte ratio could mirror the circulating immune status of the host [19]. The MLR level may be more stable than independent monocyte, lymphocyte, and leukocyte levels because of the balance between the monocyte and lymphocyte levels, which is less affected by various physiological and pathological statuses. MLR has been considered a novel inflammatory biomarker as a readily available and inexpensive index calculated by routine blood examination. Therefore, MLR might be a good reflection of the different clinical conditions in patients with DR.

Some studies have suggested that the increase in MLR may be associated with the production of pro-inflammatory chemokines such as interleukin-6 (IL-6, tumor necrosis factor, IL-1β, and monocyte chemotactic protein 1. These chemokines play major roles in the recruitment and activation of monocytes and leukocytes and the subsequent inflammatory responses in patients with DR [10, 20]. Song Yue et al. suggested that higher MLR values may be an independent risk factor for DR [21]. Huang et al. demonstrated that patients with DR patients in the proliferative stage had significantly higher MLRs than those in the non-proliferative stage, and that MLR is a powerful predictor for the occurrence of DR [20]. Our findings are consistent with those of some previous studies. We conducted this study for the first time to our knowledge with a larger sample size in the U.S. population, and focused on the association between MLR and PDR. In the present study, we found that patients with PDR had a remarkably higher MLR than those with NPDR. Because of the enhanced inflammatory response and reduced immune function, a high MLR in patients with PDR may result from an increased number of monocytes and decreased number of lymphocytes. Therefore, our findings suggest that MLR is closely associated with the risk of PDR. The pathogenesis of DR development and progression is complicated, and the role of VEGF in DR is unclear. More studies are needed to determine whether there is a correlation and mutual influence between VEGF and MLR.

Heng Wan et al. revealed that low peripheral blood monocyte levels may be a biomarker for screening at the early stages of DR, but the levels of neutrophils and lymphocytes are not necessarily associated [22]. DR may result in increases in the monocyte levels in the peripheral blood through the attraction and influx of monocytes into the retina by adhering to the outer surface of retinal capillaries and breaking down the blood-retinal barrier [23]. These controversial results may have resulted from the different conditions of the participants, such as subject heterogeneity and lifestyle differences.

This study had some limitations. First, our study is based on data from the NHANES database, making it a cross-sectional study. Even though a relationship between the MLR and the presence of PDR was established, the causal relationship could not be addressed because of the study’s cross-sectional design, further research requires a prospective study. Second, fundus photographs which based on 2-field photography were not a wide-field photography, which may have led us to a misclassification bias and an underestimation of severe DR in the United States. Finally, As some diabetes data were obtained from self-reported recalls, recall and self-reporting bias might occur. Nevertheless, given these limitations, it is essential to design a multi-center-controlled trials to verify our findings.

## Conclusions

The aim of the present study was to investigate the relationship between MLR and the occurrence of PDR. The MLR was significantly increased in T2D participants with PDR after adjusting for confounding variables. MLR is a convenient and economical biomarker derived from routine blood examination and may play an important role in follow-up visits in T2D patients. Additional studies are needed to identify the mechanism underlying the association between MLR and PDR.

## Supplementary Information

**Additional file 1.** Supplementary Tables S1–S5: Association between MLR and the presence of PDR after multiple imputations.

## Data Availability

The NHANES data analyzed in the current study are publicly available.

## Abbreviations

C-T2D: Type a diabetes
DR: Diabetic retinopathy
PDR: Proliferative diabetic retinopathy
NPDR: Nonproliferative diabetic retinopathy
MLR: Monocyte-lymphocyte ratio
NHANES: National Health and Nutrition Examination Survey
NCHS: National Center for Health Statistics
VEGF: Vascular endothelial growth factor
WBC: White blood cell
HbA1c: Glycated hemoglobin
FPG: Fasting plasma glucose
CRP: C-reactive protein level
BMI: Body mass index
HDL: High-density lipoprotein
OR: Odds ratios
CIs: Confidence intervals
