# Supplementary material for: Association of monocyte-lymphocyte ratio and proliferative diabetic retinopathy in the U.S. population with type 2 diabetes
Source: J Transl Med. 2022 May 13;20:219. doi: 10.1186/s12967-022-03425-4 (PMC9102352; doi:10.1186/s12967-022-03425-4)
Supplement: Supplementary file 1 — Additional file 1. Supplementary Tables S1–S5: Association between MLR and the presence of PDR after multiple imputations. [file 12967_2022_3425_MOESM1_ESM.docx]

| Additonal fiile 1: Tables S1. Association between MLR and the presence of PDR | | | | |
| --- | --- | --- | --- | --- |
|  | PDR(n=27) | | | |
|  | Model1 | | Model2 | |
|  | OR (95% CI) | P | OR (95% CI) | P |
| MLR*10 | 1.4 (1.11-1.75) | 0.004 | 1.46 (1.09~1.97) | 0.012 |
| Adjusted covariates: Model 1: unadjusted; Model 2: adjusted by age, sex, race; Model 3: Model 2 + HGB, duration of diabetes. MLR, monocyte-lymphocyte ratio; PDR, proliferative diabetic retinopathy; OR, odds ratio; CI, confidence interval; HGB, hemoglobin. | | | | |

| Additonal fiile 1: Tables S2. Association between MLR and the presence of PDR | | | | |
| --- | --- | --- | --- | --- |
|  | PDR(n=27) | | | |
|  | Model1 | | Model2 | |
|  | OR (95% CI) | P | OR (95% CI) | P |
| MLR*10 | 1.4 (1.11-1.75) | 0.004 | 1.44 (1.08-1.93) | 0.013 |
| Adjusted covariates: Model 1: unadjusted; Model 2: adjusted by age, sex, race; Model 3: Model 2 + HGB, duration of diabetes. MLR, monocyte-lymphocyte ratio; PDR, proliferative diabetic retinopathy; OR, odds ratio; CI, confidence interval; HGB, hemoglobin. | | | | |

| Additonal fiile 1: Tables S3. Association between MLR and the presence of PDR | | | | |
| --- | --- | --- | --- | --- |
|  | PDR(n=27) | | | |
|  | Model1 | | Model2 | |
|  | OR (95% CI) | P | OR (95% CI) | P |
| MLR*10 | 1.4 (1.11-1.75) | 0.004 | 1.45 (1.08-1.94) | 0.012 |
| Adjusted covariates: Model 1: unadjusted; Model 2: adjusted by age, sex, race; Model 3: Model 2 + HGB, duration of diabetes. MLR, monocyte-lymphocyte ratio; PDR, proliferative diabetic retinopathy; OR, odds ratio; CI, confidence interval; HGB, hemoglobin. | | | | |

| Additonal fiile 1: Tables S4. Association between MLR and the presence of PDR | | | | |
| --- | --- | --- | --- | --- |
|  | PDR(n=27) | | | |
|  | Model1 | | Model2 | |
|  | OR (95% CI) | P | OR (95% CI) | P |
| MLR*10 | 1.4 (1.11-1.75) | 0.004 | 1.46 (1.09-1.96) | 0.012 |
| Adjusted covariates: Model 1: unadjusted; Model 2: adjusted by age, sex, race; Model 3: Model 2 + HGB, duration of diabetes. MLR, monocyte-lymphocyte ratio; PDR, proliferative diabetic retinopathy; OR, odds ratio; CI, confidence interval; HGB, hemoglobin. | | | | |

| Additonal fiile 1: Tables S5. Association between MLR and the presence of PDR | | | | |
| --- | --- | --- | --- | --- |
|  | PDR(n=27) | | | |
|  | Model1 | | Model2 | |
|  | OR (95% CI) | P | OR (95% CI) | P |
| MLR*10 | 1.4 (1.11-1.75) | 0.004 | 1.44 (1.08-1.93) | 0.014 |
| Adjusted covariates: Model 1: unadjusted; Model 2: adjusted by age, sex, race; Model 3: Model 2 + HGB, duration of diabetes. MLR, monocyte-lymphocyte ratio; PDR, proliferative diabetic retinopathy; OR, odds ratio; CI, confidence interval; HGB, hemoglobin. | | | | |
